# Clinical psychology training and accreditation: Meeting demands without jeopardizing quality

**DOI:** 10.1371/journal.pmen.0000188

**Published:** 2024-11-14

**Authors:** Amit Kumar Soni, Mohit Kumar

**Affiliations:** 1 Department of Clinical Psychology, National Institute of Mental Health and Neurosciences, Bengaluru, Karnataka, India; 2 Department of Psychology, Government Maharani Laxmi Bai Girls Postgraduate College, Devi Ahilya University, Indore, India; 3 Department of Psychology, Government Prime Minister College of Excellence, Dhar, India; 4 Department of Psychiatry, All India Institute of Medical Sciences, Bhopal, India; PLOS: Public Library of Science, UNITED KINGDOM OF GREAT BRITAIN AND NORTHERN IRELAND

## Introduction

India faces a significant shortage of clinical psychologists, with only 0.47 per 100,000 people, far below the WHO’s recommended one per 20,000 [1,2]. According to the Rehabilitation Council of India (RCI), there are only 3,890 clinical psychologists and 2,896 rehabilitation psychologists nationwide [3]. To meet demand, India needs well-trained professionals capable of providing comprehensive mental health services [4]. The M.Phil. in Clinical Psychology has historically been a cornerstone of professional training in India. However, due to the discontinuation of M.Phil. programs under the National Education Policy 2020 (NEP-2020) and directives from the University Grants Commission (UGC), there are rising concerns about the future of clinical psychology education in the country [5].

**Fig 1:**
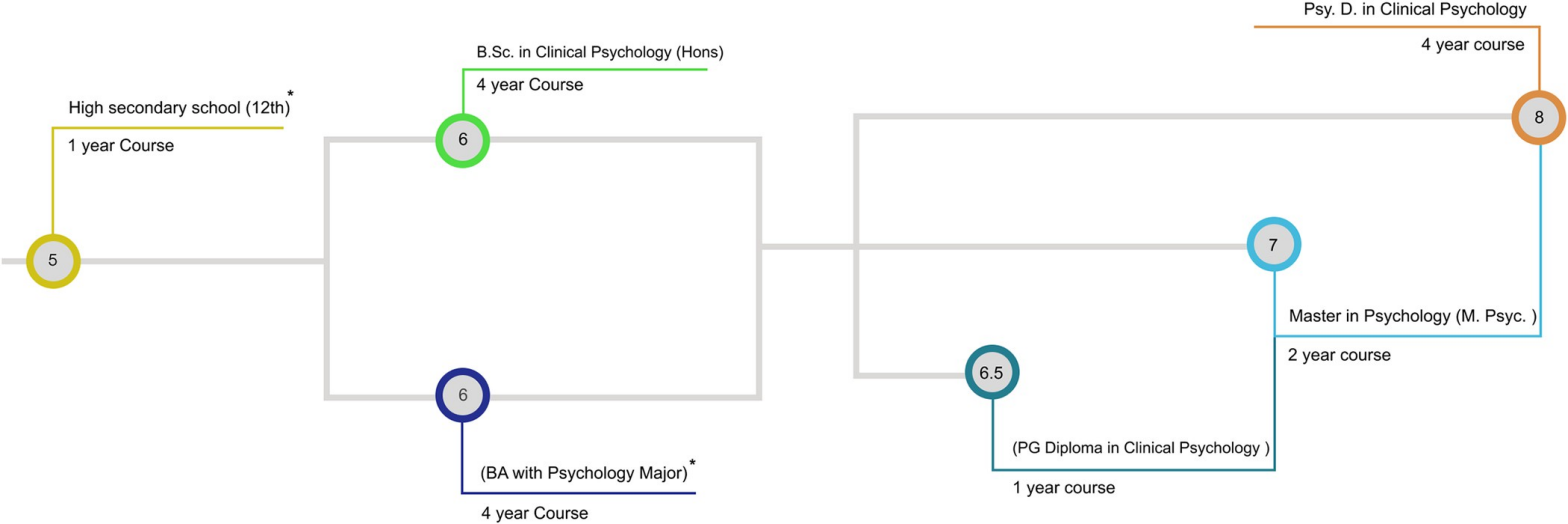
Educational Journey of clinical psychologists in India. Numbers in circles denote levels in the National Higher Education Qualifications Framework (NHEQF). * = course that are not accredited by Rehabilitation Counsel of India.

In response, the RCI introduced new accredited programs, including the B.Sc. in Clinical Psychology, Postgraduate Diploma, M.Psych., and Psy.D. While these programs offer multiple entry and exit points (Fig 1), they have resulted in confusion regarding the quality of training and the clarity of professional roles within the field [6]. The proliferation of new qualifications with less rigorous training threatens the quality of mental health services, raising key issues that need urgent attention. Indeed, this trend is not unique to India and such concerns have broader global applicability.

### Key issues and challenges

Before NEP-2020, the primary route was the two-year M.Phil. in Clinical Psychology, evolving from programs from Diplomas in Medical and Social Psychology to the M.Phil. in Medical and Social Psychology, and eventually to the M.Phil. in Clinical Psychology [7]. Over time, the RCI has introduced few accredited training programs, including the Psy.D. in Clinical Psychology, the Professional Diploma in Clinical Psychology. Despite the RCI later introducing new courses, the M.Phil. in Clinical Psychology remained the most esteemed qualification—essential for professional recognition and employment—due to its more rigorous training compared to other accredited programs. Following the discontinuation of all M.Phil. programs under NEP-2020, the RCI currently accredits four new programs for becoming a clinical psychologist, ranging from one to four years [8]. However, these new courses were introduced without clear guidelines on the discontinuation of older programs, creating confusion and disparities in professional competencies. The existence of multiple training paths for the same licensure category allows less rigorous routes to licensure, undermining the quality of training and professionalism in the field, ultimately affecting mental health services [9].

The RCI maintains the Central Rehabilitation Register (CRR), which includes 16 professional categories and provides licensure to various rehabilitation professionals, including Clinical Psychologists [3,8]. With the addition of new accredited courses, the RCI introduced new licensure categories named "Clinical Psychologist (Associate)" and "Counseling Psychologist (Mental Health)" [10], Having multiple licensure categories within the same profession adds confusion regarding roles. Poorly defined responsibilities lead to overlaps and inconsistencies, as shown in Table 1. The overlapping scopes of practice and poorly defined roles among these categories contribute to job dissatisfaction and can negatively impact the quality of services.

**Table 1:** Summary of the CRR categories, and course.

CRR Category	Old Courses	New Courses	Allowed to perform				
			Case History and Diagnosis	Psychometry	Neuropsy.	Counseling	Therapy
Clinical Psychologist	M.Phil. in Clinical Psychology	M. Psych. in Clinical Psychology; Psy.D.	Yes	Yes	Yes	Yes	Yes
Rehabilitation Psychologist	M.Phil. in Rehabilitation Psychology	?	Yes	Yes	Yes	Yes	Yes
Associate Clinical Psychologist	Professional diploma in Clinical Psychology	PG Diploma in Clinical Psychology	Yes	Yes	No	No	No
Counseling Psychologist	-	B.Sc. in Clinical Psychology (Honors)	?	?	?	Yes	?

Note: Neuropsy. = Neuropsychologial assessment and rehabilitation;? = status unknown.

Clinical psychologists are often narrowly categorized as rehabilitation professionals, despite their broader role in mental health care. Under the Mental Healthcare Act of 2017, they are recognized as mental health professionals with expertise in diagnosing and treating various psychological conditions [11]. Treating them merely as rehabilitation professionals undervalues their expertise and restricts their contributions. This narrow focus undermines their ability to address India’s diverse mental health needs, limiting the quality and scope of care. Recognizing their full skill set is crucial for improving mental health outcomes, rather than confining their roles to rehabilitation [11].

The RCI’s accreditation practices have raised concerns within the clinical psychology community. Although the RCI lists only a few accredited programs on its website [8], the actual list of qualifications registered under the CRR is much broader, including several outdated, inconsistent, non-accredited programs [3,12]. The CRR includes eight different courses for clinical psychologists, and the RCI has licensed professionals with non-accredited qualifications like MA/MSc or Ph.D. degrees in Psychology, which may not meet the rigorous clinical training requirements needed for the field [12]. This inconsistency in licensure practices undermines the credibility of the profession and erodes public trust in mental health services.

Recently, the RCI accredited the delivery of the Post Graduate Diploma in Rehabilitation Psychology (PGDRP) through open and distance learning mode [13]. This decision raises significant concerns regarding the competency of future mental health professionals, as the Clinical psychology training intensive supervised training and hands-on experience—conditions that are challenging to meet through distance learning.

### Proposed solutions

To address the challenges arising from recent changes in clinical psychology training and accreditation in India, we recommend several key solutions. First and foremost, consolidating licensure categories is essential. Globally, clinical psychology follows a standardized model, similar to psychiatry, which has one category: psychiatrist. In countries like the U.S., Canada, and Australia, clinical psychologists obtain a doctoral degree, complete supervised practice, and pass licensure exams, without "associate" roles. India’s multiple categories create confusion and dilute professional identity. Consolidating into a single "Clinical Psychologist" category with standardized qualifications like a Psy.D. or Ph.D. will ensure professionals meet rigorous standards, enhancing service quality and credibility.

Second, India should streamline accreditation to one or two pathways, such as Psy.D. or Ph.D., similar to psychiatry’s clear routes: MD or DPM. These psychiatric qualifications maintain rigorous, standardized training, ensuring no confusion. Similarly, clinical psychology programs should be limited to these options, overseen by a dedicated body of clinical psychologists. This will uphold high standards and align training with global best practices.

Third, even if multiple licensure categories are maintained, clearly defined roles and responsibilities are crucial to avoid overlap and confusion. Each category should have a distinct scope of practice, outlining specific duties and limitations. This clarity will streamline resource allocation, improve collaboration among professionals, and ensure that patients receive appropriate care. For instance, clinical psychologists should focus on the assessment, diagnosis, and treatment of mental health disorders, while counseling psychologists should specialize in providing support for life issues through counseling and guidance. Reducing redundant licensure will enhance efficiency and professionalism in the field.

Finally, India needs a unified regulatory body governed by psychologists to oversee accreditation, licensure, and professional standards. This body could integrate organizations like the Indian Association of Clinical Psychologists (IACP) and the Clinical Psychologist Society of India (CPSI) under the Government of India. Such an organization would ensure that all clinical psychologists adhere to high ethical and professional standards, and it would streamline regulatory processes. A centralized regulatory body would also help in monitoring continuing education, providing licensure renewals, and enforcing disciplinary actions when necessary. Countries like the US, Canada, and Australia have implemented similar unified systems with success [11].

## Conclusion

India, like many parts of the world faces significant challenges in training and accrediting clinical psychologists, impacting the quality of mental health services. By streamlining categories to a single "Clinical Psychologist" standard, limiting pathways to high-standard qualifications such as Psy.D. or Ph.D., and establishing a unified regulatory body, countries would ensure that professionals meet rigorous standards. This approach would not only improve the quality of mental health services but also reinforce professional training, integrity and accountability, elevating the standards of mental health care delivery Meeting the demands for Clinical Psychologists in India should not be met with workarounds that hinder the quality of training, thus potentially harming those using the services.
